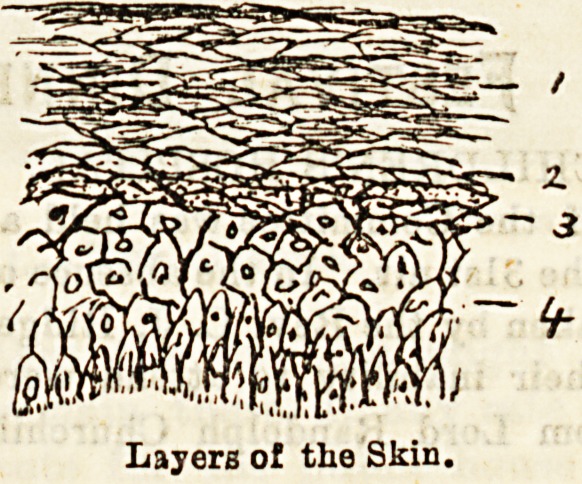# The Methods and Applications of Skin Grafting

**Published:** 1893-06-10

**Authors:** 


### LONDON HOSPITAL.

### The Methods and Applications of Skin Grafting.

Among the resources of modern surgery there are few operations simpler in their performance but more striking in their results than skin grafting in its various forms. Perhaps no other measure within the range of the surgeon is of more use in the prevention or removal of deformity, or gives a better loophole of escape from the law of nature, seeing that, where the whole thickness of the skin is destroyed, contraction must inevitably take place, and with it necessarily more or less deformity. The application of skin grafting in surgery has a wide field of usefulness, and one whose limitations are as yet but dimly seen, the recent application of Thiersch's method of grafting to the treatment of malignant growths having enlarged an area of usefulness that before seemed defined and limited.

The methods of grafting used at this hospital vary according to the nature of the case, and may be shortly placed under three headings:—

(1) The application of small pieces of skin, either human or from a frog.

(2) The application of large pieces of human skin from one part of the body to another.

(3) Thiersch's method of skin grafting.

The first of these methods is the simplest and most often used. The *modus operandi* is as follows.—

The skin, preferably of the arm, being cleansed, a small piece, about equal in area to a small grain of wheat, is pinched up with forceps and cut off with one snip of a sharp pair of scissors. If it has been pinched up carefully the cut will not have gone through the whole thickness of the true skin, and bleeding will be very slight. Should the patient be very nervous, or object much to the pain, a few drops of 2 to 5 per cent. solution of cocain may be injected beneath the skin at the spot from where the grafts are to be taken, and does not interfere with the vitality of the removed skin.

Application of small Skin Grafts to an Ulcer.

The small graft is now placed on a microscope slide or other small piece of glass with a few drops of boracic lotion or normal saline solution (a ¾ per cent. solution of common salt), and cut with a sharp scalpel or razor into three or four still smaller bits. These are placed without delay on the surface to be grafted, care being taken that the raw surface of the small piece of skin is placed next the surface it is grafted on. Instead of using human skin, frog's skin is occasionally used, and answers equally well. The frog is well washed in water to remove the slime and dirt, placed under a glass with a few drops of chloroform, which quickly kills it, or pithed, that is to say, a stout needle or fine probe passed into the brain and moved about so as to destroy consciousness. Larger pieces of skin can be removed than from the human arm, taking only skin, and not any of the subjacent tissues, cut up in the same way as described above into bits about half the size of a wheat grain and placed on the surface to be grafted. The efficiency of this method depends more on the number of grafts used than on their size, a large graft being less likely to take than three or four smaller ones.

The conditions under which this method of skin grafting is useful are wide and varied, and may be roughly classified into three groups: (1) Surfaces left after burns, deep enough to destroy the true skin, and which if left to themselves would either never heal or heal with great contraction and deformity; (2) small ulcers; (3) wounds and injuries involving skin that have to be allowed to heal by granulation, as, for instance, a crush of a limb in machinery accidents where a large strip of skin has been torn or crushed off.

The surface to be grafted has to undergo a certain amount of preparation before it is fit for the reception of grafts, the granulations on its surface must be small and healthy, nor must there be much discharge of pus or serous fluid. This condition can usually be obtained by the judicious use of wet astringent dressings and boracic fomentations, either singly or alternating with each other; the most generally useful method being to clean the surface by means of boracic fomentations for a few days, then tone down the exuberant granulations by means of wet dressings of sulphate of zinc (one to four grains to ounce), or nitrate of silver (two to four grains to ounce), finally, for twenty-four hours before grafting, to again use boracic fomentations to bring about an increased vascular supply to the part.

The surface having been gently washed with either normal solution, boracic lotion, or freshly boiled water, the grafts are applied raw surface down, with a fine forceps or needle. Lines of grafts are best placed about half-an-inch apart, starting from the point of the surface that under ordinary circumstances would be the last to be reached by the ingrowing skin, also putting a ring of grafts round the margin of the granulating surface, where their presence seems to stimulate the growing margin of epidermis to increased efforts. It is not advisable to try and do too much at one time, and cover too large an area, thirty or forty grafts being the maximum that it is advisable to use at any one time. The surface is now dusted with finely powdered dry boracic acid, and a piece of green protective, larger than the grafted surface by at least half-an-inch in every direction, applied. Sometimes the boracic acid powder is omitted, and is certainly not an essential, though if it has not been possible to get the surface quite free from tendency to discharge fluid (pus or serum), the powdered boracic seems to give the grafts a better chance of retaining their position. A layer of cotton wool is lightly applied over the protective, and the whole secured with a soft bandage, splints being used if possible to ensure complete rest of the parts.

The dressings are not touched for three days, when they are carefully removed, the surface washed clean of pus with a very gentle stream of warm boracic lotion. Most of the grafts will be either almost or completely invisible. The superficial layer of the epithelium in all cases loosens and comes away, and until the deeper layers of the graft begin to grow it is occasionally difficult to be certain of the exact spot where they have been applied. A few days later, when the new cells have grown, they are easily detected, a thin greyish pearly margin of new epithelium surrounding each, and spreading over the granulating surface.

The part is redressed in the same way for another three or four days, when, if it be preferred, boracic or other non-irritating ointment may be substituted for the protective.

If the extent of the surface be such as to require it, a fresh series of grafts may be applied in the course of a week or ten days.

Grafting by Second Method to Ulcer left after contraction of a Burn.

The second method of skin grafting—the transference of a larger piece of skin from one part of the body to another, is equally useful in suitable cases, though not being applicable to such a wide range of cases. As it has been found that a large piece of skin, say over an inch square, dissected off, and transplanted to another part, rarely if ever lives, the procedure has been modified by taking the flap from an area of good skin close to the part where the graft is wanted, dissecting it off everywhere except at one point, where it is left connected by a neck of skin. The main body of the graft can now be twisted, so as to fit into the place where it is required, and sutured into position with fine silk or silkworm gut sutures, the neck of skin being left for a week or ten days, when the graft will have taken growth in its new position, and will no longer need the blood supply through the connecting bridge of skin that ensured its vitality till it had formed fresh connections in its new position.

The graft is cut so that its long axis is parallel to the long axis of the limb. The edges of the wound left by its removal then easily come together with sutures, and being in the length of the limb the skin easily stretches, and no appreciable contraction takes place.

The spot where it is intended to place the graft is prepared for its reception by having the callous edge of skin round its margin dissected off, and its base freshened by a little judicious dissection, all bleeding being carefully stopped before the graft is sutured in position. The graft is then dressed with some antiseptic dressing for ten days, when the graft will be found to have taken, the sutures removed, and the neck of skin connecting the graft with its original site cut through.

This method is particularly applicable to those cases of burns, or injuries on the face or limbs, where, from either the situation or extent of the injury complete cicatrization never takes place, or, if it does, repeatedly breaks down again when the limb is moved. Of course, it is a necessity that there be some sound skin in the immediate neighbourhood, from which a graft can be taken.

Layers of the Skin.

The third method is that introduced by Thiersch, and called after him. It consists in shaving off with a razor—the cut not going through the whole thickness of the skin—pieces several square inches or less in area from the arm or thigh, and applying them to ulcers or any other raw surface that may require covering with skin.

It is necessary here to glance briefly at the structure of the skin to arrive at an intelligent comprehension of the performance of this method. The skin may be taken for our purpose as consisting of four layers named from without in (1) stratum corneum, (2) stratum lucidum, (3) stratum granulosum, (4) stratum malpighii. The deepest, or malpighian, layer consists of true living cells, which become more and more flattened out as they are pressed towards the surface, lose their vitality, till by the time they have arrived at the two outermost layers they are no longer living, but dead or dying scales, protecting the deeper living layers, and awaiting their turn to be cast off as scales or scurf.

The grafts in Thiersch's method must pass deep enough to include the stratum granulosum, or, better still, the most superficial cells of the deepest layer in order to ensure that living cells will be taken with the graft, to grow and multiply in their new position.

These grafts take best if applied to a fresh, raw surface, better than on granulation tissue, so that when they are used for ulcers the edges of the ulcer are trimmed away, and the whole of the granulation tissue of the floor dissected off, leaving a fresh surface, to which, after all bleeding has been stopped, the grafts are applied.

Not only in ulcers and burns, but also in malignant growths, is this method of signal service. A cancer of the breast may require such free incision to get clear of the diseased tissue that the margins of the wound cannot be brought together. The part instead of being left to heal over slowly by granulation is covered with skin grafts by this method, and is skinned well over by the time the dressings are changed. Especially in rodent cancer of the face is Thiersch's skin grafting useful, in the prevention of unsightly deformity. The cancer being dissected completely off, and bleeding stopped, the whole of the raw surface is covered with skin grafts, and heals often with a scarcely noticeable scar.

The grafts are taken as a rule from the thigh or upper part of the arm, the skin being thoroughly cleansed beforehand, strong antiseptics being avoided while the grafting is being done, as they tend to impair the vitality of the living cells of the transplanted graft, either boracic lotion or normal saline solution being used to wash out the wound before the grafts are applied.

The wound may be dressed either with protective and cotton wool or any of the ordinary antiseptic gauzes, redressed in four or five days, when a homogeneous layer of new growing skin will be found over the whole extent of the wound.

The parts from which the grafts are taken should not bleed; if more than oozing of blood-stained serum occurs, the cut has gone deeper than is necessary; new epidermis soon forms in place of that removed, the parts being kept comfortable by a dressing of boracic ointment or gauze. It is, perhaps, scarcely necessary to add that this form of grafting, being extremely painful while the grafts are being cut, should be always done under an anæsthetic.

## Figures and Tables

**Figure f1:**
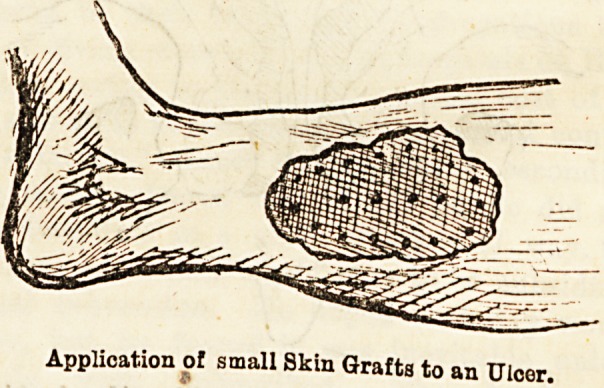


**Figure f2:**
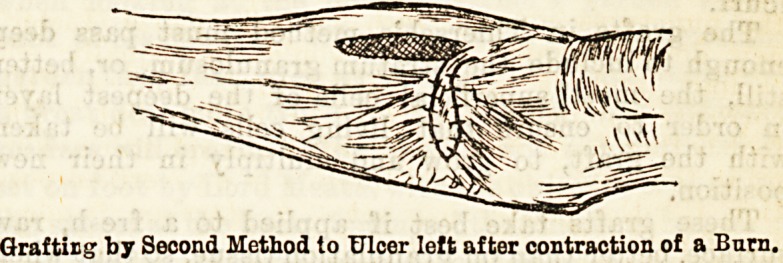


**Figure f3:**